# Ancient genomes reveal a high diversity of *Mycobacterium leprae* in medieval Europe

**DOI:** 10.1371/journal.ppat.1006997

**Published:** 2018-05-10

**Authors:** Verena J. Schuenemann, Charlotte Avanzi, Ben Krause-Kyora, Alexander Seitz, Alexander Herbig, Sarah Inskip, Marion Bonazzi, Ella Reiter, Christian Urban, Dorthe Dangvard Pedersen, G. Michael Taylor, Pushpendra Singh, Graham R. Stewart, Petr Velemínský, Jakub Likovsky, Antónia Marcsik, Erika Molnár, György Pálfi, Valentina Mariotti, Alessandro Riga, M. Giovanna Belcastro, Jesper L. Boldsen, Almut Nebel, Simon Mays, Helen D. Donoghue, Sonia Zakrzewski, Andrej Benjak, Kay Nieselt, Stewart T. Cole, Johannes Krause

**Affiliations:** 1 Institute for Archaeological Sciences, University of Tübingen, Tübingen, Germany; 2 Senckenberg Centre for Human Evolution and Palaeoenvironment, University of Tübingen, Tübingen, Germany; 3 Institute of Evolutionary Medicine, University of Zurich, Zurich, Switzerland; 4 Global Health Institute, Ecole Polytechnique Fédérale de Lausanne, Lausanne, Switzerland; 5 Institute of Clinical Molecular Biology, Kiel University, Kiel, Germany; 6 Department of Archaeogenetics, Max Planck Institute for the Science of Human History, Jena, Germany; 7 Center for Bioinformatics, University of Tübingen, Tübingen, Germany; 8 McDonald Institute for Archaeological Research, University of Cambridge, Cambridge, United Kingdom; 9 Unit of Anthropology (ADBOU), Department of Forensic Medicine, University of Southern Denmark, Odense S, Denmark; 10 Department of Microbial Sciences, Faculty of Health and Medical Sciences, University of Surrey, Guildford, United Kingdom; 11 Department of Microbiology and Biotechnology Centre, The Maharaja Sayajirao University of Baroda, Vadodara, India; 12 Department of Anthropology, National Museum, Prague, Czech Republic; 13 Department of Archaeology of Landscape and Archaeobiology, Institute of Archaeology of the Czech Academy of Sciences, Prague, Czech Republic; 14 Department of Biological Anthropology, University of Szeged, Szeged, Hungary; 15 Department of Biological, Geological and Environmental Sciences, Bologna, Italy; 16 ADES AMU-CNRS- EFS: Anthropology and Health, Aix-Marseille Université, Marseille, France; 17 Department of Biology, University of Florence, Firenze, Italy; 18 Historic England, Portsmouth, United Kingdom; 19 Centre for Clinical Microbiology, Division of Infection and Immunity, University College London, London, United Kingdom; 20 Department of Archaeology, University of Southampton, Southampton, United Kingdom; 21 Institut Pasteur, Paris, France; Stanford University School of Medicine, UNITED STATES

## Abstract

Studying ancient DNA allows us to retrace the evolutionary history of human pathogens, such as *Mycobacterium leprae*, the main causative agent of leprosy. Leprosy is one of the oldest recorded and most stigmatizing diseases in human history. The disease was prevalent in Europe until the 16^th^ century and is still endemic in many countries with over 200,000 new cases reported annually. Previous worldwide studies on modern and European medieval *M*. *leprae* genomes revealed that they cluster into several distinct branches of which two were present in medieval Northwestern Europe. In this study, we analyzed 10 new medieval *M*. *leprae* genomes including the so far oldest *M*. *leprae* genome from one of the earliest known cases of leprosy in the United Kingdom—a skeleton from the Great Chesterford cemetery with a calibrated age of 415–545 C.E. This dataset provides a genetic time transect of *M*. *leprae* diversity in Europe over the past 1500 years. We find *M*. *leprae* strains from four distinct branches to be present in the Early Medieval Period, and strains from three different branches were detected within a single cemetery from the High Medieval Period. Altogether these findings suggest a higher genetic diversity of *M*. *leprae* strains in medieval Europe at various time points than previously assumed. The resulting more complex picture of the past phylogeography of leprosy in Europe impacts current phylogeographical models of *M*. *leprae* dissemination. It suggests alternative models for the past spread of leprosy such as a wide spread prevalence of strains from different branches in Eurasia already in Antiquity or maybe even an origin in Western Eurasia. Furthermore, these results highlight how studying ancient *M*. *leprae* strains improves understanding the history of leprosy worldwide.

## Author summary

Many controversies surround leprosy, which is one of the oldest recorded diseases of humankind. The origin and past spread of its main causative agent, *Mycobacterium leprae*, remain unknown although many attempts have been made to reconstruct its past from historical and archeological sources. Analysis of ancient *M*. *leprae* genomes reconstructed from archaeological remains can contribute greatly to reconstructing the origin and evolution of this pathogen. With a new set of ancient *M*. *leprae* genomes from Europe, we traced back a so far unrecognized past diversity, which places Europe as a key region for the early spread and worldwide dissemination of leprosy. Our results hint to the potential dynamic changes in the prevalence of different *M*. *leprae* strains in Europe during Antiquity, and highlight the need to study ancient pathogen genomes in order to better understand our past.

## Introduction

Leprosy resulting from the infection with *Mycobacterium leprae* has been prevalent since early history. Signs of its existence occur in historical texts [1, 2] as well as in osteological and archeological records [3–5]. Widespread in medieval Europe, and peaking between the 12^th^ and 14^th^ century, leprosy declined in the 16^th^ century and subsequently disappeared from Europe [6, 7]. Nowadays *M*. *leprae* is prevalent worldwide except in Europe and is genetically represented by distinct branches with different geographic distributions [8]. *M*. *leprae* branches correspond to specific single nucleotide polymorphism (SNP) types or subtypes [9], a nomenclature widely used in comparative genomics, consisting of four SNP types (1–4) and 16 SNP subtypes (A to P) [10]. While SNP types are based on a limited number of SNPs [10], the classification of *M*. *leprae* into above-mentioned branches is based upon whole genome comparison and therefore reflects more complexity [9]. The most ancestral branch, branch 0, (partially corresponding to SNP subtype 3K) is mainly found in Eastern Asia (Japan, China, New Caledonia) whereas branch 1 (corresponding to SNP type 1) is mostly detected in Southern and Eastern Asia (Thailand, India, Southern Japan) [9–13]. SNP type 2, which includes branch 2, is reported in the Near East and South Asia [10, 13, 14]. Branch 3 (corresponding to SNP subtype 3I) is present in Latin America and recently spreading in the southwestern USA nine-banded armadillo population [15] with occasional zoonotic transmission to humans [16]. Other non-human *M*. *leprae* reservoirs include the red squirrel in England [17] and non-human primates in West Africa and the Philippines [18]. Finally, branch 4 (corresponding to SNP type 4) is present in West Africa and South America [9, 10, 19].

Investigations on the evolutionary history of *M*. *leprae* have elucidated the past phylogeography and diversity of the leprosy bacillus in Europe. Recently sequenced medieval *M*. *leprae* genomes reveal the presence of at least two distinct *M*. *leprae* branches in medieval Northwestern Europe [9]. Furthermore, the data indicate a high level of genetic conservation during the last 1000 years. There appears to be a close relationship of a group of late medieval strains with contemporary strains present today in the Southwestern USA [9] infecting humans and armadillos [15] as well as red squirrels in England [17]. Two medieval genomes from a cemetery in the UK suggested a possible predominance of branch 2 during the 10^th^ to the 12^th^ century in Northwestern Europe, while branch 3 was more frequent during the Late Medieval Period [20]. However, the past diversity and population structure of *M*. *leprae* at different time points in other parts of Europe still remain unclear.

To address these key questions, we sequenced four whole genomes of *M*. *leprae* strains identified in early medieval leprosy cases from various parts of Europe—Italy, Hungary, Czech Republic and the UK—including one of the oldest leprosy cases in the UK from an early Anglo-Saxon cemetery in Great Chesterford, radiocarbon dated to 415–545 C.E. [21]. Furthermore, we sequenced six genomes from the Odense St. Jørgen cemetery in Denmark to assess the diversity of *M*. *leprae* in one location at a particular point in time. Our results reveal a high diversity of *M*. *leprae* branches in early medieval Europe, where four main *M*. *leprae* branches were identified. Furthermore, three branches of *M*. *leprae* were found within the St. Jørgen cemetery alone, indicating a high level of strain diversity in medieval Europe.

## Results

### Sample collection and screening

**Fig 1:**
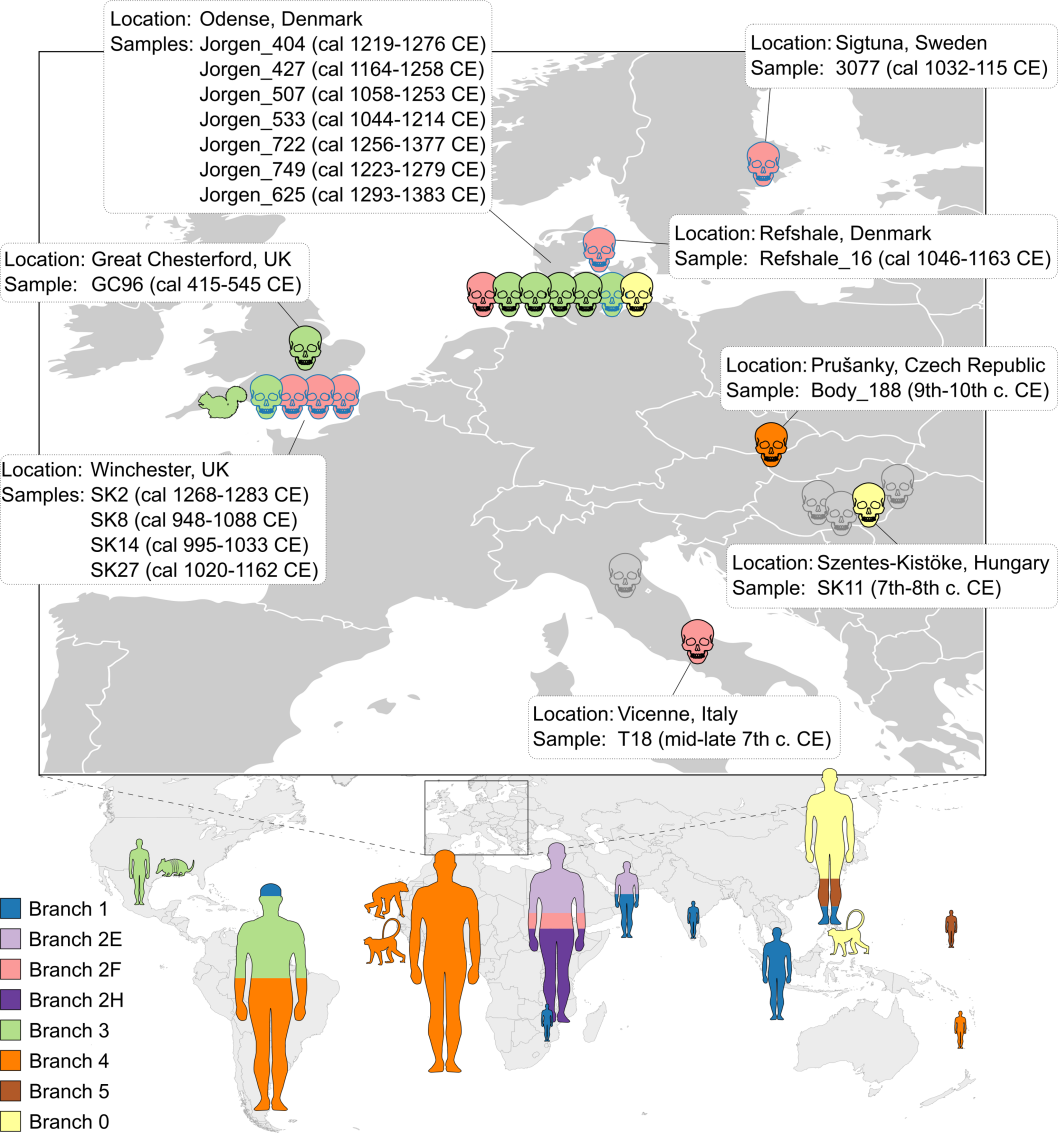
Worldwide distribution of the ancient and modern *M*. *leprae* strains analyzed in this study. Skulls represent strains from osteological specimens dated to the Medieval Period. Human silhouettes represent modern strains, sized to scale according to the number of samples, ranging from 1 (e.g. India) to 36 (South America) Animal silhouettes represent strains from the red squirrel, the nine-banded armadillo, and naturally infected nonhuman primates (a chimpanzee from Sierra Leone, a sooty mangabey from West Africa, and a cynomolgus macaque from The Philippines). Skulls outlined in black are the new *M*. *leprae* genomes reconstructed in this study, while skulls outlined in blue represent previously sequenced ancient genomes. Grey skulls are leprosy samples from this study that did not yield sufficient sequence for whole-genome analysis. The main *M*. *leprae* lineages, represented by branches (see Fig 2) are color-coded.

The potential leprosy cases analyzed in this study were identified based on characteristic skeletal deformations and span different European countries and time periods (see Fig 1 and S1 Table for details of all samples except the negative Danish ones): two from Italy (4^th^ B.C.E. and 7^th^ C.E.) [22–24], one from UK (5^th^ to 6^th^ century) [21], four cases from Hungary (7^th^ to 11^th^ century) [23], one from the territory of today’s Czech Republic (9^th^ to 12^th^ century) [23], and 87 from Denmark (11^th^-14^th^ century).

The screening for the Danish samples was carried out with PCR [9] and direct shotgun sequencing of double-stranded Illumina libraries [25, 26] followed by metagenomics analysis using MALT [27]. Samples with more than 1% (n = 6, S1 Table) of all raw reads mapping to the *M*. *leprae* TN reference genome (RefSeq ID NC_002677.1) were used for whole genome sequencing by shotgun sequencing without any prior enrichment. For all other samples double-stranded Illumina libraries [25, 26] were created and screened for *M*. *leprae* DNA using a bead capture approach [28] on three genomic loci, *gyrA*, *proS* and *RLEP*, as detailed previously [9], yielding between 0.4x and 700x-fold average coverage for the enriched genes. DNA misincorporation patterns characteristic for ancient DNA were calculated to assess the authenticity of the retrieved DNA [29, 30]. As observed previously [9], ancient *M*. *leprae* DNA contained a lower percentage of misincorporation patterns, between 11 and 21% (S1 Table), compared to what is expected for human DNA of the same region and age [31]. This consistency underlines the exceptionally good long-term preservation of *M*. *leprae* DNA within cells as already commented on previously [9].

### Genome-wide enrichment, sequencing, and analysis

On the basis of the screening results (S1 Table), samples containing *M*. *leprae* DNA were subjected to whole-genome enrichment and sequencing. The libraries were prepared using an enzymatic DNA damage repair [32]. Prior to sequencing, all libraries were enriched using an array spanning the *M*. *leprae* genome [9, 33], except for the Danish samples, which were directly shotgun sequenced. Between 406,241 and 12,227,587 short reads were mapped to the *M*. *leprae* TN reference genome (RefSeq ID NC_002677.1) using the EAGER pipeline [34] and all samples with at least 7x-fold mean coverage were selected for further analysis (Tables 1, S2). For the resulting 10 samples, we used the Genome Analysis Toolkit (GATK) to generate a mapping assembly to call reference bases and variants from the mapping.

**Table 1:** Results of the genome-wide analysis for the samples with sufficient coverage.

Sample Name	Mapped non-duplicate reads	Mean Coverage	Genome fraction covered by at least 5 non-duplicate reads	average fragment length	SNPs per sample
Jorgen_404	394594	10.8	98.97	89.65	87
Jorgen_427	1087657	29.8	99.98	89.65	54
Jorgen_507	1997693	59.8	99.99	97.84	183
Jorgen_533	378098	11.1	98.07	95.61	108
Jorgen_722	385329	11.2	98.44	94.56	118
Jorgen_749	1353305	40.1	99.99	96.92	91
GC96	777067	20.5	97.38	86.21	96
T18	329362	7	61.32	69.59	25
Body_188	3561241	116	99.99	106.45	101
SK11	442725	7.8	68.99	57.51	45

### Phylogenetic analysis

**Fig 2:**
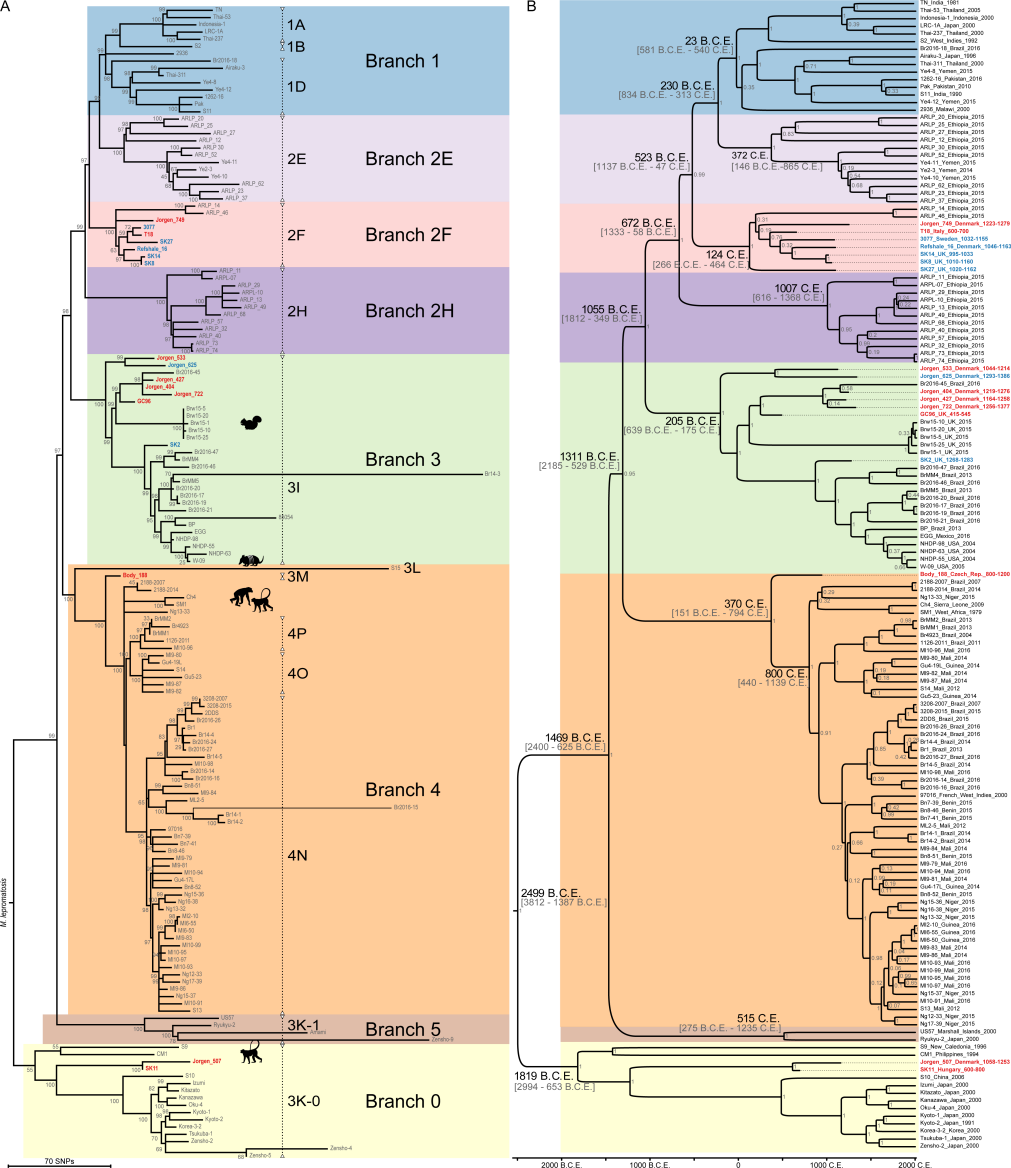
Phylogenetic analysis of ancient and modern *M*. *leprae* strains. (**A**) Maximum parsimony tree reconstructed from 3124 informative SNP positions. The tree is drawn to scale, with branch lengths representing number of substitutions. *M*. *lepromatosis* was used as outgroup. The novel strains from this study are labelled in red, and the previously published ancient strains are labelled in blue. Animal symbols indicate strains isolated from red squirrels, armadillos and non-human primates. The main branches are color-coded, and the SNP subtypes are marked with dotted vertical bars. Bootstrap values (500 replicates) are shown next to each node. (**B**) Bayesian phylogenetic tree based on 2371 SNP positions calculated with BEAST 1.8.1. Median divergence times in years B.C.E. and C.E. are shown on the main nodes (the 95% Highest Posterior Density ranges are given in square brackets). Tip labels for each sample show the name, the country of origin and the isolation date, or the radiocarbon dates. The novel strains from this study are labelled in red, and the previously published ancient strains are labelled in blue. Posterior probabilities for each node are shown in grey. The main branches are color-coded. The hypermutator strains 85054, Amami, S15, Br14-3, Br2016-15, Zensho-4, Zensho-5 and Zensho-9 (as described in [14]) were excluded from this analysis.

We reconstructed the phylogeny for the 10 newly sequenced and well-resolved medieval genomes together with the previously sequenced seven medieval [9, 20] and 142 modern genomes [8, 9, 11, 14–19], from a total of 3124 SNP positions. The inclusion of the newly published genomes from Benjak and colleagues [14] resulted in three additional branches in the *M*. *leprae* phylogeny compared to the earlier study with fewer genomes [9]. We named the new monophyletic ancestral branch as branch 5 (Fig 2A), while the paraphyletic branches corresponding to SNP type 2 were named 2E, 2H and 2F (the former branch 2 [9]) (see Fig 2A). The four early medieval genomes fall on different branches in the phylogeny (Fig 2A). The oldest *M*. *leprae* genome from Great Chesterford (GC96) belongs to branch 3, as do the armadillo, red squirrel, and other medieval and modern human strains [9, 17].

The medieval genome from Italy (T18) falls within branch 2F, whereas SK11 from Hungary clusters with the branch 0 strains. The Body188 strain from Czech Republic belongs to branch 4 and is ancestral to contemporary strains from Western Africa. The six individuals from the St. Jørgen cemetery in Denmark, which was established in 1270 and existed until 1560, are 14C-dated with overlapping periods and mean values from the 12^th^ to the 14^th^ century (S1 Table), a period in which the majority of burials took place [35]. The six *M*. *leprae* genomes obtained from these individuals cluster within three different branches (Fig 2A): branch 0 (n = 1), branch 2F (n = 1) and branch 3 (n = 4).

### SNP analysis

We analyzed the 3124 SNP positions identified in the whole dataset for the effects that they might have on genes (S3 Table). No polymorphisms specific only to all ancient strains were found. Non-synonymous SNPs (nsSNPs) specific to each branch are given in S4 Table.

### BEAST analysis

Using the radiocarbon dates for the ancient samples and the isolation dates for the modern samples as tip calibration points we estimated the divergence time for all *M*. *leprae* strains using BEAST [36] assuming a constant population size [37] and a variable population size by application of a Bayesian Skyline model [38]. The age of the most recent common ancestor (MRCA) was estimated to 4,515 y BP (3,403–5,828 y 95% Highest Posterior Density (HPD)) under the constant population size model (Fig 2B), and 4,031 y BP (3,110–5,020 y 95% HPD) assuming the variable population size (Bayesian Skyline) model (Table 2). The estimated mean age of the MRCA is about 1,000–1,500 y older compared to the dating analysis previously performed [9] with fewer modern day and medieval genomes available. The shift was mainly due to the ancient genomes from branch 0, which show a higher variability, compared to other branches. However, the 95% HPD intervals of both analyses overlap, increasing the confidence of the estimates. The age of the MRCAs of the main branches varied between 3,835 y BP (2,669–5,010 y 95% HPD) for branch 0 and 1,009 y BP (648–1,400 y 95% HPD) for branch 2H (see Table 2). The mutation rate for *M*. *leprae* was estimated to 7.36 x 10^−9^ (5,72–9.11 x 10^−9^ 95% HPD) and 7.53 x 10^−9^ (6.05–8.97 x 10^−9^ 95% HPD) substitutions per site per year for constant and variable population size, respectively. This rate can be also depicted as 18–30 mutations per 1000 years and genome.

**Table 2:** Time to the most recent common ancestor (tMRCAs) for the entire *M*. *leprae* tree and individual branches (HPD = Highest Posterior Density).

	constant pop. size		Bayesian Skyline	
Branch	mean tMRCA [y]	95% HPD [y]	mean tMRCA [y]	95% HPD [y]
**whole tree**	4515	3403–5828	4031	3110–5020
**0**	3835	2669–5010	3326	2443–4270
**5**	1501	781–2291	1415	785–2051
**1**	2039	1476–2597	1895	1489–2331
**2E**	1644	1150–2162	1592	1148–2005
**2F**	1891	1551–2282	1815	1543–2122
**2H**	1009	648–1400	1004	697–1377
**3**	2221	1841–2655	2097	1787–2440
**4**	1646	1222–2167	1612	1233–2023

## Discussion

The 10 newly sequenced medieval *M*. *leprae* genomes allow us to trace back the last 1500 years of leprosy history in Europe and illustrate the high diversity of strains circulating in Europe during this time transect. Previous genome wide data suggested the existence of only two branches in medieval Europe, branch 2F (previously branch 2) present at least from the 10^th^ to the 12^th^ century and branch 3 in the Late Medieval Period [9, 20]. Our results reveal a higher diversity in medieval Europe than anticipated. Strains belonging to the ancestral branch 0 and associated with modern strains from East Asia were found in Hungary (SK11) and Denmark (Jorgen 507) confirming the presence of this branch in Europe since at least the 7-8^th^ century [23]. The so far oldest *M*. *leprae* genome, GC96 from Great Chesterford falls into branch 3 confirming the existence of this branch in Europe since at least the 5^th^ century. Interestingly, the *M*. *leprae* strains isolated from red squirrels from England [17] are placed tightly between GC96 from Great Chesterford (5^th^-6^th^ century), SK2 from Winchester (11^th^ century) and three strains isolated from the St. Jørgen cemetery in Denmark (Jorgen_722, Jorgen_427 and Jorgen_404) (10^th^-13^th^ century) (Fig 2), corroborating the hypothesis that the red squirrel was infected with a strain that was circulating in humans in medieval Europe at the time. Given the current impact of the zoonotic spread of leprosy in the Americas through the nine-banded armadillo [15, 16], it is not impossible that contact with red squirrels was a contributing factor in the spread of the disease in the past. Indeed, historical sources demonstrate that there was a strong European squirrel fur trade, particularly in Baltic squirrels, which were imported into the East Anglian part of Britain in the Medieval Period [39]. Moreover, squirrels were hunted for food, and often used as pets. For example, squirrels are commonly depicted wearing collars and leashes in medieval art and they were mentioned in historical letters as pets in monasteries [40]. The keeping of squirrels in the domestic space could be envisioned as a possible mechanism for a transfer of *M*. *leprae* between species. Interestingly, the ability of *M*. *leprae* to infect animals is not restricted to this particular branch, as exemplified by the strains isolated from naturally infected nonhuman primates belonging to branch 4 and 0 [18] (Fig 2).

With the T18 strain we detected the branch 2F also in early medieval Central Italy, while until now it was found only in Northern Europe [9, 20]. The Body188 strain from Czech Republic on the other hand is ancestral to modern branch 4 strains from West Africa and Brazil. This strain belongs to the SNP subtype 3M, which has not yet been sequenced and is rarely identified in modern samples [10]. Based on our phylogenetic tree we can now group SNP subtype 3M together with modern SNP-type 4 strains into branch 4 (Fig 2A). Body188 provides therefore a link between Europe and West Africa, where the contemporary SNP-type 4 is predominant [10].

The dynamics of *M*. *leprae* transmission throughout human history are not fully resolved, but characterization and geographic association of the most ancestral strains are crucial for deciphering leprosy’s origin, which still remains elusive. This is in part due to the scarcity of convincing evidence of leprosy in historical records that predate the Common Era [41]. The earliest accepted written record of leprosy is in the Sushruta Samhita, an old Indian text on medicine and surgery dated around 600 B.C.E. [1], and with the exception of a limited number of probable cases based on paleopathological evidence, such as the so far oldest one from India dated around 2000 B.C.E. [3] or cases from Italy and Hungary dated to the 4^th^-3^rd^ century B.C.E. [22, 42]. However, all are not yet confirmed on the molecular level. The oldest osteological cases of leprosy that could be detected by molecular methods are from around the beginning of the Common Era [10, 23], when written records also become more abundant [2, 41]. Therefore, at present, we are limited to molecular methods to decipher the time of *M*. *leprae*’s origin and its early spread in humans. Having more ancient genomes in a dating analysis will result in more accurate estimates. In this study, almost every major *M*. *leprae* branch was represented by at least one ancient genome. The estimated mean age of the MRCA is about 700–1000 y older compared to the estimates derived from fewer medieval genomes [14], and about 1,000–1,500 y older compared to the estimates derived from fewer modern and medieval genomes [9]. The shift probably resulted from the higher genetic variability in the larger datasets. Nevertheless, the estimates from this study had a remarkable overlap with the previous estimates, which is not surprising and reflects the robustness of using century-old genomes for dating a relatively young and conserved population. The new dating results indicate that the *M*. *leprae* population is at least a few thousand years old, justifying the search for even older osteological cases of leprosy than currently available, using well-established methods for identification of potential cases [43].

The high diversity of *M*. *leprae* in medieval Europe, spanning almost all its major branches including the most basal ones in the phylogeny, is rarely found elsewhere. Exceptions, like New Caledonia for example [10], might include remote regions where leprosy was likely introduced from multiple sources. On the other hand, regions harboring *M*. *leprae* for a long time are expected to display an elevated genomic diversity assuming there were no bottlenecks. Based on these observations two antipodal models can be developed to explain the high diversity of *M*. *leprae* in Europe: an origin of leprosy in Western Eurasia, maybe even in Europe, and spread from there into the rest of the world (model 1), and the introduction of *M*. *leprae* strains from diverse branches to Europe from different regions in the world during and before the Medieval Period (model 2). The main argument supporting both models is that Europe has long had features favorable to the spread of infectious diseases such as leprosy. From at least the Bronze Age, trade networks connected different parts of the continent [44], which first became especially extensive in the 1^st^ and 2^nd^ century C.E. during the height of the Roman Empire. Cities, which provide the high populations densities that are favorable to infectious diseases, are also a feature of the European landscape since the Iron Age [45]. Altogether, the dynamic geopolitical changes, wars and the well-established trading routes contributed to continuous contacts with the neighboring regions and migrations within and outside Europe and allow fast and multiple exchanges of pathogens in both directions. In favor of model 1 (Western Eurasian origin) is the argument that most medieval European *M*. *leprae* strains are ancestral to modern strains (Fig 2). On the other hand, supporting model 2 (multiple introductions into Europe) is the prevalence of the most ancestral branch 0 (subtype 3K) in modern day China, Japan and Korea [10, 14, 46–48] and in the Middle East [10]. Unfortunately, the lack of ancient and modern genomic data for the key regions, like Central Asia or the Near East, do not allow us to favor one model over the other. Overall, the current genomic data would fit both models and convincingly suggest Eurasia as a broad area of origin and of the early spread of *M*. *leprae*. Including more ancient strains from different parts of the world in a phylogenetic context may enable us to resolve this issue and trace back a narrower region of the origin, although this can be challenging for areas where past events have become blurred by extensive population mixing, bottlenecks or strain replacements. Furthermore, neither model entirely conflicts with the osteoarcheological evidence so far, or to some degree with historical sources. The oldest osteoarcheological cases from India [3] and Europe [22, 42] could prove very valuable for deciphering the history of leprosy, if only genomic data could be successfully retrieved from them.

It is not clear whether the main *M*. *leprae* branches differ in virulence. Current epidemiological data show that all branches can cause different forms of leprosy [47, 49]. Although no study to date has investigated this matter closely, in absence of obvious epidemiological patterns, and considering that *M*. *leprae* acquires on average only between 18–30 mutations per 1000 years as calculated in this study, we can deduce that virulence and transmissibility do not dramatically differ between the different branches, or at least not enough to have a strong short-term effect. We showed that in Europe, different branches of *M*. *leprae* were simultaneously circulating during the same period of time, spanning several centuries. Moreover, all the European medieval strains have modern relatives (Fig 2) that are currently spreading elsewhere. Therefore, the sudden decline of leprosy in Europe 500 years ago is probably not due to a loss of bacterial infectiousness or virulence but was more likely caused by human cultural or biological adaptation or by environmental factors (successive pandemics such as cholera or tuberculosis followed by hygiene improvements). Previously, Donoghue and colleagues [23] already suggested a scenario of a combination of population movements from the land to the emerging cities in Europe triggered by the social consequences of the Black Death, increasing urbanization and a large rise in the incidence of tuberculosis leading to tuberculosis killing leprosy patients. Furthermore, the feasibility of the scenario was supported by an epidemiological analysis [50].

In summary, our results provide a genetic time transect for the diversity of *M*. *leprae* within the Medieval Period in Europe and allow us to gain a better understanding of the past phylogeography of this pathogen. Discovery of other ancient *M*. *leprae* strains, especially from Asia, will provide new details on the diversity of *M*. *leprae* in the past and help develop models for its global spread.

## Methods

### Ancient DNA extraction and library preparation

DNA extractions from the samples JK3187 to JK3195, GC96F and GC96C (S1 Table) were conducted from 30–50 mg bone powder for each sample in clean room facilities dedicated to ancient DNA work at the University of Tübingen. The Danish samples were processed separately at the University of Kiel following the same protocols as described below for the samples processed in Tübingen. A silica purification protocol was applied as previously described [51] using the following modifications: the Zymo-Spin V columns (Zymo Research) were UV irradiated for 60 minutes and the total elution volume was raised to 100 µl.

Aliquots of 20 µl from JK3187 to JK3195, GC96F and GC96C were converted into double-stranded Illumina libraries [25]. The adapter-ligated fragments were quantified through a quantification assay using the primers IS7 and IS8 [25], the DyNAmo Flash SYBR Green qPCR Kit (Biozym) and the Lightcycler 96 (Roche).

Following established protocols [25, 26] sample-specific indexes were added in the next step to both library adapters via amplification to create double indexed libraries. Extraction and library blanks were treated accordingly. These libraries were used subsequently for the initial screening approaches.

For genome-wide enrichment and sequencing additional libraries were prepared from 30 to 50 µl aliquots of all DNA extracts according to the methods described above [25, 26] with one modification: One additional step- the treatment of all extracts and blanks with uracil-DNA glycosylase (UDG) and endonuclease VIII- was included into library preparation to avoid potential sequencing artefacts caused by the characteristic ancient DNA damage profile produced by the deamination of cytosine to uracil over time [32].

For all indexed libraries a subsequent amplification was performed as detailed in Schuenemann and colleagues [9].

### Enrichment and sequencing for sample screening

All samples except the Danish ones were screened for *M*. *leprae* preservation. Three *M*. *leprae* genes—ML0006 (*gyrA*), ML1553 (*proS*) and RLEP- were selected as targets for DNA enrichment and converted into bait DNA using Long Range PCR products as described previously [9]. Following the bead enrichment protocol by Maricic and colleagues [28] a hybridization of the amplified libraries, pooled in an equimolar amount, to the DNA bait was carried out.

Subsequently to the bead enrichment the libraries were sequenced on an Illumina HiSeq 2500 platform using a paired-end dual index run with 2*101+8+8 cycles (for the samples GC96F and GC96C) and for 2*125+8+8 cycles (for the samples JK3187 to JK3195) following the manufacturer’s protocols for multiplex sequencing (TruSeq PE Cluster Kit v3-cBot-HS).

The screening for the Danish samples was carried out with PCR for parts of specific genes (18kDa antigenic protein gene and repetitive element RLEP gene) [9] and direct shotgun sequencing of double-stranded Illumina libraries [25, 26]. The sequencing for the shotgun data was performed on the Illumina HiSeq 4000 platform at the Institute of Clinical Molecular Biology, Kiel University, by 2×75 cycles using the HiSeq v4 chemistry and the manufacturer’s protocol for multiplex sequencing.

### Genome-wide enrichment and sequencing

The UDG treated libraries (one from JK3187 to JK3195, GC96F and two from GC96C) were enriched genome-wide with two rounds of hybridization capture following the protocol detailed before [52]. The design of the 1 million Agilent SureSelect arrays used in the study was described previously [9]. In a first approach equimolar pools of GC96F and GC96C1 were enriched on one array, in a second experiment equimolar pools GC96C1 and GC96C2 on two arrays. In a third approach the samples JK3187 to JK3189 and JK3192 to JK3195 were enriched on one array and JK3190 and JK3191 on a separate one. In all three experiments the extraction and library blanks were enriched separately on an additional array. After the first round of hybridization, captured products were eluted in 490 µl H_2_O and quantified via a quantitative PCR with the IS5 and IS6 primer set [25], the DyNAmo Flash SYBR Green qPCR Kit (Biozym) and the Lightcycler 96 (Roche). In a subsequent amplification the eluted products were amplified for 17 to 20 cycles in 100 µl reactions using 24 µl template, 4 units of AccuPrime Pfx DNA polymerase (Invitrogen), 1 unit of 10× AccuPrime buffer (containing dNTPs) and 0.3 µM of the primers IS5 and IS6 [25] and the following thermal profile: a 2-min initial denaturation at 95°C, 17 to 20 cycles consisting of 15 sec denaturation at 95°C, a 30-sec annealing at 60°C and a 2-min elongation at 68°C, followed by a 5-min final elongation at 68°C. The amplified enriched library pools were purified using MinElute columns (Qiagen) following the manufacturer’s protocol and quantified via an Agilent 2100 Bioanalyzer DNA 1000 chip. All pools were then enriched in a second round of hybridization capture using the same number of arrays as in the first round. After the second round the capture products were eluted and subsequently processed as previously described with the following modification: 48 µl template was used per amplification reaction.

After the enrichment paired-end dual indexing sequencing was performed on an Illumina HiSeq 2500 platform using 2*101+8+8 cycles (for the samples GC96F and GC96C) and for 2*125+8+8 cycles (for the samples JK3187 to JK3195) using the manufacturer’s protocols for multiplex sequencing (TruSeq PE Cluster Kit v3-cBot-HS). A second round of sequencing was conducted for the samples JK3187 to JK3195 on an Illumina HiSeq 4000 platform using a single-end dual index run with 75+8+8 cycles.

### Genome-wide sequencing for the Danish samples

The sequencing of the six Danish samples was carried out on the Illumina HiSeq 4000 platform at the Institute of Clinical Molecular Biology, Kiel University, by 2×75 cycles using the HiSeq v4 chemistry and the manufacturer’s protocol for multiplex sequencing.

### Screening analysis

The data processing after screening for all samples was carried out as previously described [9] with modifications of using the EAGER pipeline [34]. For all samples except the Danish ones the processed reads were mapped to the three *M*. *leprae* loci *gyrA*, *proS*, and *RLEP* and characteristic damage profiles were calculated for the *M*. *leprae* DNA to assess the authenticity of the ancient DNA [29, 30].

For the Danish samples a metagenomics analysis using MALT [53] was conducted after the processing through the EAGER pipeline [34]. In addition, the shotgun data was also mapped to the *M*. *leprae* TN reference genome (RefSeq ID NC_002677.1).

### Genome-wide analysis

The sequenced reads from all samples subjected to genome-wide enrichment were analyzed with the EAGER pipeline [34].

#### Read preprocessing of sequenced genome samples

The first step of the pipeline was adapter clipping, read merging (for pair-end datasets), and subsequent quality trimming using the tool "Clip&Merge". For all properly merged reads, only the merged consensus sequence was used for the subsequent mapping steps. On average about 89% of all paired-end reads were merged in each sample (see S2 Table for detailed results of all samples). For those read pairs that could not be merged because the overlap region was shorter than 10 nucleotides or for which the corresponding read was removed during the combined adapter clipping and quality filtering step, the respective single-end reads were first trimmed at the 3’end such that all bases have a Phred quality score of at least 20 and then mapped individually.

#### Mapping

After adapter clipping, merging and quality trimming, the resulting reads for all samples were mapped using the *M*. *leprae* TN genome (RefSeq ID NC_002677.1) as a reference. All reads (merged and unmerged) were treated as single-end reads and mapping was performed using BWA [54] aln/samse subcommands, with an error rate (-n) of 0.2 to assure high specificity. The PCR duplicates were removed with MarkDuplicates from the Picard tools (https://broadinstitute.github.io/picard). The mapping was evaluated with QualiMap [55].

#### Mapping assembly

After mapping and duplicate removal, the Genome Analysis Toolkit (GATK) [56] was used to generate a mapping assembly for each sample that had at least 60% genome coverage and a minimum of 5 reads per base. For this procedure the UnifiedGenotyper module of GATK following the GATK Best Practice’s Guidelines was applied to call reference bases and variants from the mapping. The reference base was called if the genotype quality of the call was at least 30, the position was covered by at least 5 reads and at least 90% of the bases at this position agreed with the reference. A variant position (SNP) was called if the following criteria were met: i) the position was covered by at least 5 reads; ii) the genotype quality of the call was at least 30 and iii) the minimum SNP allele frequency was 90%. If neither of the requirements of a reference base call nor the requirements for a variant call were met, the character ‘N’ was inserted at the respective position. To keep the potential introduction of too many ‘N’ characters as low as possible in the case of low coverage genomes, in cases where a position had a coverage between 5–9 reads, the major allele was called if it was found in all but 1 read. Positions covered in a negative control [9] were excluded from subsequent phylogenetic analyses.For the generation of draft genome sequences we used the VCF2Genome tool, also available in EAGER.

#### Processing of published modern genomes

The reads for the modern samples *S2*, *S10*, *S11*, *S13*, *S14*, and *S15*, which were previously published [9], were single-ended. Thus, these samples were not merged but only adapter-clipped and quality trimmed. Afterwards, the reads were treated exactly the same as the other samples. In order to apply our analysis pipeline also to those samples for which complete genomic sequences are available in GenBank (*Br4923*, and *TN*), we produced artificial reads using an in-house tool (Genome2Reads). In a tiling approach, we produced reads of length 150 nucleotides with a tiling offset of 2, resulting in an average genome coverage of 75X. For the resulting samples we applied the same mapping, SNP calling and genome reconstruction procedure as for the sequenced samples in order to obtain consistent and comparable results. The same was done with the genome sequence of *Mycobacterium lepromatosis* (GenBank JRPY00000000.1) to be used as an outgroup for the maximum parsimony tree.

#### Phylogenetic analyses

For the phylogenetic analysis, a SNP alignment based on 3124 informative positions was generated. This alignment contained all positions where a SNP was called in at least one sample. Positions covered in a negative control [9] were excluded from subsequent phylogenetic analyses. From the resulting alignment of length 3124 bp a phylogenetic tree was created with MEGA7 [57] using the Maximum parsimony method. All positions with less than 80% site coverage were eliminated, resulting in a total of 3092 positions in the final dataset. The bootstrap test was done with 500 replicates.

### Dating analysis

We estimated divergence times and substitution rates by application of the Bayesian framework BEAST 1.8.1[38]. In this analysis based on 2371 SNP positions we included all ancient and modern strains (Fig 2) except for the hypermutator strains 85054, Amami, S15, Br14-3, Br2016-15, Zensho-4, Zensho-5 and Zensho-9 (as described in [14]). We performed two analyses using a constant population size coalescent prior [37] and a Bayesian Skyline model [38] for variable population size, respectively. For both analyses we applied a lognormal relaxed clock and an HKY substitution model. For ancient strains tip dates were uniformly sampled from dating intervals [9, 20, 21, 23] (samples from this study S1 Table) whereas for modern strains tip dates were set as isolation dates [9–11, 15–17, 19]. For each model, an MCMC run was carried out with 300,000,000 iterations discarding the first 30,000,000 iterations as burn-in.

### Ethical statement

Research on human historical remains presents the ethical problem that informed consent can no longer be obtained from the individuals themselves. In almost all cases it is furthermore impossible to trace down close relatives that would be able to provide an informed consent to conduct genetic or anthropological research of their ancestors. As the historical human remains used in this study are over 100 years old, they are not subject to the Human Tissue Act (2004). We are however not using culturally sensitive material such as Native American- or Australian Aboriginal populations, where disturbance of burial grounds raises culturally sensitive ethical concerns. The samples described in the manuscript have been previously excavated throughout Europe, some of the excavators are among the authors of the manuscript. In addition, most sites represent rescue/salvage excavations and were uncovered due to ongoing construction. Our research is furthermore not motivated by questions relating to population affinity, but instead we focus on infectious diseases that potentially affect all human populations regardless of cultural identity.

Permission to the medieval human analyzed in this study remains were granted by the Odense Bys Museer, National Museum of the Czech Republic, University of Bologna, University of Szeged, English Heritage / Historic England and University of Southampton.

Further details on the collections are provided below:

The Odense St. Jørgen skeletons were excavated by Odense Bys Museer in 1980–1981, and they are now stored at the Unit of Anthropology (ADBOU), Institute of Forensic Medicine, University of Southern Denmark. The Museum has approved the sampling of the skeletons for this study.

The skeleton from Great Chesterford was excavated in the 1950s by the Inspectorate of Ancient Monuments in response to skeletons accidentally being found during gravel extraction in the area. Prior ethical approval has been obtained for research using these remains from English Heritage / Historic England and University of Southampton.

As regards the Italian medieval skeletons of Vicenne-Campochiaro (CB, Molise, Italy) they were excavated around 1990 by the Soprintendenza Archeologia Belle Arti e Paesaggio del Molise (sezione Archeologia). The Soprintendenza agreed to bring all the skeletons of the Vicenne necropolis into the Laboratory of Bioarchaeology and Forensic Osteology of the Department of Biological, Geological and Environmental Sciences of the University of Bologna (Italy), where they are still, for anthropological study purposes.

The Szentes Kistőke skeleton from Szeged was excavated by Gábor Csallány, archaeologist of Szeged Museum in 1931. The skeletal remains are now stored in the collection of the Department of Biological Anthropology, University of Szeged, Hungary. The use in this study was approved by the University of Szeged.

The skeleton from medieval Prušánky burial site was excavated in the years 1978–1980 by the Institute of Archaeology of the Czech Academy of Sciences in Brno. Subsequently the skeletons from this burial site were stored in the Department of Anthropology of the National Museum in Prague, which have approved the sampling of the skeleton for this study.

### Accession numbers

The data for the four early medieval strains (GC96, T18, Body188, and SK11) is deposited in the NCBI SRA Archive with the BioProject ID PRJNA417381 and the data for the six St. Jørgen genomes can be found in the European Nucleotide Archive with the study accession number ERP021830.

## Supporting information

S1 Table Details of the samples and screening results. (XLSX)

S2 Table Details of the results of the genome-wide analysis. (XLSX)

S3 Table Details of the SNPs per individual sample. (XLSX)

S4 Table Branch-specific protein-changing SNPs. (XLSX)
